# Assessment of Acceptability, Usage, and Impact on Caregivers of Children With Autism’s Stress and Mindfulness: Multiple-Method Feasibility Study of the 5Minutes4Myself App’s Mindfulness Module

**DOI:** 10.2196/54171

**Published:** 2024-10-31

**Authors:** Elizabeth Larson, Rebecca L Mattie, Sophia A Riffkin

**Affiliations:** 1 Occupational Therapy Program, Department of Kinesiology, University of Wisconsin-Madison, 2180 MSC, 1300 University Avenue, Madison, WI, 53706, United States, 1 608-265-0520; 2 Honeybee OT, LLC, Private Practice, Onalaska, WI, United States; ^3^University of Wisconsin-Madison, Madison, WI, United States

**Keywords:** autism, caregiver, activities, mindfulness, mobile application, stress, wellness, app, application, usage, children, developmental disability, usability, acceptability, meditation, wellness application

## Abstract

**Background:**

Caregiver wellness programs need to be easily accessible to address caregivers’ constraints to participation.

**Objective:**

We aimed to assess the feasibility of 5Minutes4Myself app’s mindfulness module (usability, usage, and impact on caregivers’ levels of mindfulness and perceived stress).

**Methods:**

Before and after participation in the 5Minutes4Myself program, 15 participants were asked to complete the Perceived Stress Scale (PSS) and Five Facet Mindfulness Questionnaire (FFMQ). Data on the usage of app-delivered meditations were collected electronically via the app, and app usability was rated on the Modified System Usability Scale. Analyses assessed participants’ frequency of use of app-delivered meditations, app usability, and changes in participants’ stress and mindfulness post intervention.

**Results:**

Overall, participants completed 10.9 minutes of mindfulness meditations per week and rated the app 76.7, indicating above-average usability. Related samples *t* tests (2-tailed) found that group PSS (*t*_10_=1.20, *P*=.26) and FFMQ (*t*_10_=−1.57, *P*=.15) pre- or postintervention mean scores were not significantly different. However, a visualization of pre- and post-PSS and mindfulness scores suggested there was a group of responders who had decreased stress with increased mindfulness. This was confirmed via an individual change analysis. The effect size of the FFMQ scores (*d*=0.47) suggests there may be treatment effects with a larger sample. A hierarchical multiple regression analysis examined the degree mindfulness impacted perceived stress; 20% of the variance in participants’ perceived stress could be attributed to increases in self-rated mindfulness (*P*=.04) when controlling for preintervention stress levels.

**Conclusions:**

Caregivers found the app highly usable and on average used low-dose levels of mindfulness meditations (10 min/wk). For responders, increased mindfulness was related to stress reduction to population-based levels.

## Introduction

Wellness programs are critically needed for the many parent caregivers of children with autism spectrum disorder (ASD) who experience increased levels of stress, poorer health outcomes, and decreased quality of life compared to parents of children with other developmental disabilities or typical development [1-6]. Caring for a child with ASD has been found to have a significant impact on the caregivers’ overall well-being [7,8]. More than half of mothers of children with ASD report mental health problems, a substantially higher percentage than the general population [5]. Time-intensive care demands that remain high over time, challenging child behaviors, and social stigma limiting participation in community activities increase the need for wellness promotion while at the same time make participation more difficult [5,8,9]. Caregivers often put their child and family’s needs before their own self-nurturing and wellness-promoting activities [10]. Ideally, wellness programs for caregivers should be tailored to be widely available to the most caregivers and address common caregiving constraints that could limit participation such as lack of time and difficulties with attending regular programming and using effective evidence-based interventions.

Evidence suggests that mindfulness practices bolster wellness for caregivers. These programs emphasize acceptance of life circumstances and loving-kindness toward oneself and others despite challenges [11]. Multiple delivery formats, including individual, group, and self-guided mindfulness trainings, have been shown to be more effective than other stress reducing interventions for caregivers of children with ASD and other developmental disabilities [12-16]. Researchers have focused on designing mindfulness program features that support these parents’ ability to participate consistently over time to provide much needed mental health and wellness benefits. Rayan and Ahmad [17] modified a mindfulness-based intervention (MBI) program for parents in Jordan [13]. The modified program was shortened to 5-weeks and included 33 hours of training, phone follow-up sessions, and text message reminders of assignments and support. Their quasi-experimental study (n=104) found caregivers of children with ASD who participated in the MBI intervention had significantly reduced stress compared to a control group. With their modified MBI, attrition rates decreased [13,17].

However, likely due to the intensity and time demands of caregiving and the occurrence of unexpected life events, some caregivers find it difficult to attend in-person mindfulness programs that require weekly in-person time commitment and travel to community sites [12,18,19]. Harnessing the usefulness of mindfulness practices for parents may require developing an alternative to the longer in-person mindfulness programs that may overcome some of the lifestyle and time barriers these caregivers experience.

Mobile platforms offer greater flexibility for delivering self-guided meditations compared to in-person or phone options. Mobile phone texts have thus far been used in this population to extend interventions delivered in in-person training sessions [17]. Tailored delivery to times and places that fit caregivers’ lifestyles, may make mindfulness intervention more convenient and accessible and cost-effective [20,21]. Digital interventions that allow personalization, goal setting, and accountability may promote caregivers’ participation in wellness promotion and reduce social health inequalities [22,23]. Given the significant need and current evidence for the usefulness of mindfulness approaches, we developed, through a collaborative recursive process with and for caregivers, a mobile app to create a more user-friendly and accessible mindfulness module for the 5Minutes4Myself caregiver wellness program [24].

This analysis used feasibility study data and was intended to examine the app usability and who may have benefitted from program participation by investigating whether there were changes in stress and mindfulness, specifically focusing on this new element of app-delivered mindfulness meditations. We aimed to examine the app’s usability, caregivers’ usage of mindfulness content, and the effect of mindfulness meditations on participants’ postparticipation levels of mindfulness and perceived stress. We recognized that other components of the 5Minutes4Myself wellness program may also have influenced perceived stress levels. We expected that (1) the app as designed with and by caregivers would be rated as acceptable, (2) the caregiver-tailoring and habit-building features of the app would encourage regular use of meditations at least twice weekly over 4 months’ time, and (3) using the micromindfulness meditations of the modified 5Minutes4Myself wellness program would increase caregivers’ mindfulness and reduce self-rated stress.

## Methods

### Study Design

This study examined the acceptability and usability of the smartphone app and its mindfulness meditations using a quasi-experimental mixed-methods design with pre- and postintervention assessments.

### App Development

The 5Minutes4Myself wellness program was developed with and for caregivers of children with ASDs. We designed a collaborative research-informed approach. First in a focus group, we shared the best available evidence for wellness promotion and occupational science principles for lifestyle change with caregivers. Occupational sciences’ core construct is that what we do each day (ie, how we “occupy” our time) matters, and how we balance our participation in a range of daily activities or occupations with different experiences can support or undermine our quality of life [24]. To create changes in our wellness requires leveraging and sustaining strategies to revise our often entrenched and comfortable daily routines. The evidence-based program principles and elements presented to caregivers focused on “doing” (eg, activities that promote pleasure or social connection) and lifestyle change strategies to achieve changes, “thinking” (eg, positive psychology practices) and “goal visioning” [24]. Second, we elicited caregivers’ feedback on these principles and elements, confirmed caregivers’ preferences and developed additional goal-visioning activities as requested. Third, we delivered the program. It included a lifestyle consultation to develop goals using motivational interviewing (MI), coaching over 4 months to pursue wellness goals, and “doing” content delivered via an iPad (eg, knitting tutorials, yoga, meditation, and activity trackers). Last, we evaluated the program in a postparticipation focus group.

After completing the program, the key change participants requested was the development of a mobile phone app to deliver mindfulness and other content that would be available to be used anytime and anywhere [24]. Further refinements caregivers requested to meet their needs and limited capacities to participate in a wellness program were to eliminate “doing” content, which was not well used on iPads, and add guilt-free goal accountability check-ins, goal profiles to remind them of their selected wellness goals and rewards to reinforce their efforts. Due to their frequent fatigue and limited technology experience, caregivers asked for simple app navigation such as prompts that quickly redirected them to the next mindfulness meditation in the series, without having to remember where they left off, and intuitive navigation between screens, and calming color schemes. Given the request for a smartphone app, we polled the caregivers on their current technology and internet access. Due to their financial circumstances not all caregivers had smartphones, and many had older versions, and not all had home access to the internet. We recognized these would be issues that needed to be addressed in the app design.

**Figure 1.**
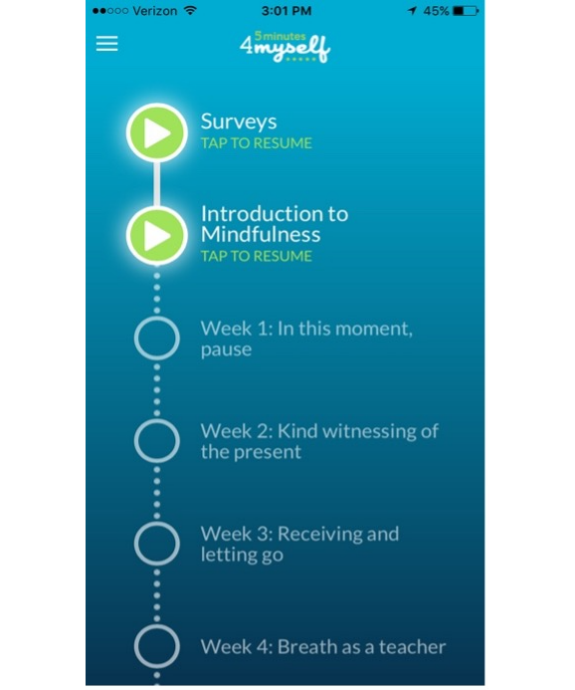
Mindfulness meditation stream screen with glow highlighting current progress.

We worked with a local software start-up company to develop both an iOS and Android app version similar in content and design, and with an expert mindfulness-based stress reduction (MBSR) trainer or clinical psychologist to develop mindfulness content. We provided the software developers with several design and function parameters tailored for our caregiving participants: aesthetically pleasing interfaces, simple navigation, offline availability of content and reminders, usability on older phones, easy personalization of goal and notification reminders, dashboard tracking of mindfulness meditation usage, request coaching help function, goal check-ins, guilt-free check-ins, and rewards for full or partial goal success. The software development team used Test Flight (Apple Inc) to deliver beta versions to our research team for testing and provided a link to a data dashboard that hosted meditation usage data and goal check-in submissions. The psychologist developed and recorded the mindfulness meditations that were specifically tailored for a caregiver. For example, loving-kindness meditations often included directing nonjudgmental and accepting thoughts toward themselves and their child. Recorded meditations were 4‐21 minutes long (Table 1). The meditation series began with an introduction to mindfulness, and then alternated between meditations emphasizing awareness of the present, being aware of the physical body, and loving-kindness. For ease of navigation, the mindfulness meditations were presented in a vertical stream with a glowing dot highlighting the next meditation to be listened to (Figure 1).

**Table 1.** 5Minutes4Myself app-delivered meditations.

Title	Content	Length (min)
Introduction of mindfulness	Introduction to program and instructor	11
In this moment, pause	Being present	3
Kind witnessing of the present	Body scan	7
Receiving and letting go	Loving-kindness	5
Breath as a teacher	Being present	6
Awake with awareness and presence	Body scan	18
Resting in the breath	Loving-kindness	10
Simply choose to pause	Being present	6
Dropping into relaxation	Body scan	22
Release, letting go	Loving-kindness	16
Resting in the moment	Breath awareness	17
Kind witnessing of the present	Body scan	8
Resting in the breath	Breath awareness	10
Deep breathing	Breath awareness	12
Awake with awareness and presence	Body scan	18
Releasing, letting go	Loving-kindness	16
Dropping into relaxation	Body scan	22

In total, 2 additional cohorts were delivered the 5Minutes4Myself program and confirmed the usefulness of these app elements (goal profile, self-timed reminders to do wellness activities, goal check-ins, goal achievement rewards, mindfulness meditations, and design or graphics) and requested navigation changes (ability to go forward as well as backward in the meditation stream) and moving goal check-ins to an opening screen [24]. Revisions were made to the app between these 2 cohorts to address app instability and remove the request for coaching support feature that was not used.

### Participants

#### Recruitment

Participants were recruited through email solicitation, posters, flyers, and brochures in the community and at conferences for families of children with ASD. Recruitment also occurred through social media advertising to ASD-specific groups. Interested individuals were screened by phone interview to assure they met inclusion criteria. Informed consent was obtained from all individual participants prior to participation at focus groups or in individual meetings. In total, 15 individuals in cohorts participated in 4-month cycles, over 1 year’s time. This sample was considered sufficient for proof-of-concept to assess the feasibility of the mobile app.

#### Inclusion and Exclusion Criteria

Caregivers were included if they were the primary caregiver of at least one child with a diagnosis of ASD aged between 8‐21 years. We selected this age grouping with the assumption that families had established care routines and thus the caregiver had more capacity to alter their daily routines for wellness promotion. Caregivers had to have a desire to participate in a lifestyle wellness program, including a mindfulness component, and be willing to commit sufficient time to participate in the program. Exclusion criteria included (1) parenting a child younger than 8 or older than 21 years of age, or (2) a caregiver diagnosis of significant mental illness such as schizophrenia, bipolar disorder, schizoaffective disorder, pervasive developmental disorder, obsessive-compulsive disorder, panic disorder, posttraumatic stress disorder, or an eating disorder. We included participants who had or were being treated for depression since clinical or subclinical levels are highly prevalent in this caregiving group [25]. In total, 5 reported being currently treated for depression. Further, 40% (6/15) of the caregivers scored 16 or greater on the Center for Epidemiologic Studies Depression Scale Revised, suggesting they were at risk for depression (mean 17, range 8‐43). A recent meta-analysis suggests that the use of stand-alone mindfulness practices, without the additional readings and instruction typically provided in MBSR programs, has been found to reduce depression and anxiety [26] thus the program could have benefits for caregivers with these diagnoses.

### Measures

#### Overview

Ratings of the app usability, app usage, and pre- and postintervention measures of self-reported stress and mindfulness were used to examine app acceptability and the effects of the 5Minutes4Myself smartphone mobile app micromindfulness meditations on caregivers’ mindfulness and stress. Measures used included the Modified System Usability Scale (MSUS), Perceived Stress Scale (PSS), Five Facet Mindfulness Questionnaire (FFMQ), and app meditation usage data. In addition, participants completed a survey that asked: their age, self-identified race or ethnicity, marital status (single, married, separated, or divorced), education (high school, associate, undergraduate, or graduate), average family income, children’s names and ages, caregiving or work (full-time caregiver, part-time work, or full-time work), medical conditions and medicines taken. This information was gathered to understand the family resources and caregivers’ health. Participants self-identified as White.

#### Modified System Usability Scale

The MSUS, a 10-item survey that yields a single score of overall usability, has been widely used to assess technology quality and functionality [27,28]. It assesses effectiveness, efficiency, and user satisfaction [26-28]. While items are rated on a 5-point Likert scale, they are weighted so that the final scores range from 0‐100 [26]. It is reliable, with *α*=.91-.92 and with good concurrent validity (*α*=.806) [26]. Scores of 68 are considered above average usability, and scores of 80.3 or greater are in the top 10% of MSUS scores [28]. Participants are asked to rate 10 statements such as “I thought the system was easy to use” or “I felt very confident using the system” from 1=strongly disagree to 5=strongly agree.

#### App Usage

To assure participants were using the meditations, data were collected via the app on the time spent listening to meditations and the number of meditations completed during the program. This allowed us to examine the dosage of mindfulness with accuracy.

#### About the PSS

The PSS is a reliable and valid self-report measure of stress (weak to moderate association criterion validity *r*=0.11-0.67, internal consistency *α*=.60-.91, and test-retest reliability *r*=0.55-0.86) [29-31]. The PSS items ask participants to rate the degree to which they have experienced feelings and thoughts of unpredictability, uncontrollability, and overload in the last month. PSS captures both daily challenges and major events within the prior 30 days. Further, 10 questions measure the degree an individual appraises their life situations as stressful using a 5-point Likert scale from “0” (never) to “4” (very often). For example, participants rated statements such as “In the last month, how often have you felt difficulties were piling up so high that you could not overcome them?” or “In the last month, how often have you felt nervous and stressed?” Higher total scores on the PSS indicate more stress [29]. For this study, the PSS measured the self-reported level of stress experienced by the participants in the month before beginning and during the last month of participating in the 5Minutes4Myself wellness program. The 10-item version was used.

#### About the FFMQ

The FFMQ is an adequately valid, reliable, and internally consistent self-report measure of mindfulness practice in an individual’s life (*α*=.73-.91, n=376) [32-34]. The 39-item measure addresses 5 characteristics that have been identified as integral to mindfulness practice including: describing, observing, acting with awareness, nonjudging of inner experience, and nonreactivity to inner experiences. Questions are rated on a 5-point Likert scale ranging from “1” (never or very rarely) to “5” (very often or always). Items include “I find it difficult to stay focused on what’s happening in the present,” “I tell myself that I shouldn’t be thinking the way I’m thinking,” and “When I have distressing thoughts or images, I judge myself as good or bad, depending on what the thought/image is about.” Higher total scores represent higher levels of mindfulness [32]. In the context of this study, FFMQ measured the degree of participants’ mindfulness before and during the last month of participating in 5Minutes4Myself wellness program which included using the smartphone mobile app mindfulness podcasts.

### Procedures

#### 5Minutes4Myself Wellness Program

All cohorts completed equivalent procedures. The 5Minutes4Myself wellness program began with each cohort attending a community-building focus group where participants discussed what worked and did not work in their lives and completed health and well-being surveys using the mobile phone app. Participants unable to attend focus groups completed surveys using the mobile app at another time, or on paper and mailed in results (this was necessary due to an Android version app failure for downloading preintervention surveys). Next, each participant was assigned a coach trained in MI; the fidelity of MI use is reported elsewhere [35]. MI is a practice in which use of collaboration, evocation, and privileging participant autonomy are essential to eliciting participant-desired lifestyle change and supporting goal attainment [36]. Participants worked with coaches to select goals meaningful to them in the initial lifestyle consultation and created a plan for instituting the goals into her or his daily life over the 4-month program. Participants identified 3 to 5 goals, which included 1 goal of regular weekly mindfulness practice. The mindfulness goal was required of participants for this feasibility study since it was a key element of the application being developed. Coaches meet at least monthly with participants, either in person or on the phone, to discuss their goal progress and revise strategies as needed.

Postintervention focus groups were conducted after 4 month’s participation. Caregivers again discussed what worked or did not work in their lives, were queried about their view of the program elements, and completed the same surveys as pre intervention as well as the MSUS. For this analysis, we used data from the MSUS, PSS, and FFMQ.

#### Smartphone Mobile App

Participants were trained to use the 5Minutes4Myself mobile app to access mindfulness meditations, to create their goal profile, to program goal reminders at preferred times, and to report weekly progress on goals via an electronic check-in. It was loaded onto participants’ personal phones, either in Android or iOS format or if needed onto a loaned Android smartphone capable of operating in a wireless environment (no phone service was provided). The audio-recorded meditations were voiced by the licensed psychologist who developed them and ranged in length from 4‐21 minutes, sequenced to increase in length over time. These began with a brief introduction to mindfulness concepts and were sequenced and available 1 per week; the focus of each meditation rotated through breath awareness, loving-kindness, and body awareness (body scans). Participants were free to choose when and where they listened to the offloaded meditations, available without an internet connection. Participants chose how often they wanted to be reminded of each of their goals: weekly, using a “guilt-free” check-in, participants for each goal included the mindfulness goal. After completing the designated week’s meditation, the mobile app advanced to the next week’s meditation; participants could revisit past meditations but could not progress to new ones until the following week. This was designed to facilitate ease of use and require no tracking on the users’ part. After the first cohort used the app, we revised the app to address bugs and improve features for following cohorts.

#### Data Collection, Management, and Analyses

Data gathered via the 5Minutes4Myself app were transferred from a secure third-party server from the application development company to the research team and removed from company servers. The software development personnel who managed the data completed the human participant research training. Caregivers with any missing data on stress or mindfulness measures were excluded from this analysis (n=4). Quantitative data were analyzed using statistical software IBM SPSS (version 24.0, IBM Corp). Descriptive statistics including means and SDs were calculated for participant demographics, and for perceived stress and mindfulness practice pre and post intervention. To contextualize the level of stress experienced, participants’ perceived stress scores were compared to a population-based mean for the PSS for their age group [29].

To explore mental health outcomes of the feasibility study, related samples *t* tests (2-tailed) were used to evaluate whether participants’ stress scores as a group (PSS) were statistically different after participating in the wellness program. A second related samples *t* test evaluated scores of mindfulness (FFMQ) before and at the end of the intervention to determine whether they were significantly different for the group. Next, to understand who benefitted from the program, we visualized the trends in these 2 measures from pre and post intervention. Responders were defined as having decreased PSS scores post intervention. In addition, to examine individual level change, an analysis was conducted using Norman and colleagues’ [37,38] approach that considers clinically meaningful change as at or above half a SD of the initial group mean. After standard mean differences are calculated ($d=\frac{D_{i}}{Sx}$ where D_i_ is individual change, S_x_ is SD of group at baseline) they are grouped into “improved” (*d*≥0.50), “no change” (−0.5<*d*>0.5), and “worsened” (*d*≤−0.50). Lastly, a hierarchical multiple regression was conducted to examine whether the pre-post change in mindfulness (change in FFMQ scores) explained variance in postparticipation perceived stress scores while controlling for preintervention stress levels with an α level of .05.

### Ethical Considerations

The study was reviewed and approved by the University of Wisconsin-Madison Educational and Social/Behavioral Institutional Review Board (2015‐1004). Participants were provided an informed consent form to review, given the opportunity to ask questions, and informed of their ability to opt out at any time. Participants who signed the consent form were invited to participate. In the compiled dataset, participants were assigned numbers; a participant key was created and stored on a password-protected computer. No compensation was provided.

## Results

### Characteristics of Participants

In total, 15 participants participated in the 5Minutes4Myself wellness program. While not all participants completed all 3 of the coaching check-ins, program completion was deemed to be participation in the initial focus group or an introductory session, completion of the lifestyle consultation, and at least 2 of 3 monthly coaching sessions. Using this metric, 13 caregivers completed the 4-month 5Minutes4Myself program for a participation rate of 87% (13/15; 2/15, 13% dropout). Further, 2 participants were excluded from the analysis due to incomplete postintervention survey responses, which were due to a family emergency and scheduling conflict with the final focus group. Neither person responded to additional requests to complete the postintervention surveys. Additionally, 11 participants completed both pre- and postintervention surveys used in this analysis. Sample characteristics and pre- and postintervention survey scores for mindfulness (FFMQ) and stress (PSS) are presented in Table 2. In this convenience sample of participants, most self-identified as White women, were married, reported caregiving full-time, were raising 2 children (one or more with an autism spectrum or other developmental disorder) with graduate school education.

**Table 2.** Sample characteristics and pre-postintervention scores^a^ (N=11).

Characteristics	Values
Age (years), mean (range, SD)	47.85 (36‐65, 8.46)
**Sex, n (%)**	
Male	1 (9)
Female	10 (91)
**Race, n (%)**	
White	11 (100)
Family income, median (range; IQR)	US $87,499 (<US $25,000‐$100,000+; US $50,000-$95,000)
**Education, n (%)**	
Graduate	8 (73)
Undergraduate	0 (0)
Associates	1 (9)
High school	1 (9)
**Marital status, n (%)**	
Single	1 (9)
Married	10 (91)
**Caregiver status, n (%)**	
Full-time caregiver^b^	7 (64)
**Employment status, n (%)**	
Part-time employee	3 (27)
Full-time employee	2 (18)
Number of children, mean (range, SD)	2.21 (1‐5, 1.12)
Age of child with autism spectrum disorder (years), mean (range, SD)	14.1 (9‐20, 3.69)
Baseline stress (PSS^c^), mean (range, SD)	23 (16‐29, 4.45)
Stress post intervention (PSS), mean (range, SD)	21 (13‐36, 8.23)
Baseline mindfulness (FFMQ^d^), mean (range, SD)	119.91 (90‐146, 15.49)
Postintervention mindfulness (FFMQ), mean (range, SD)	127 (93‐153, 17.71)

a One participant provided only partial demographic information.

b Full-time caregiver indicates major or sole responsibility for child’s care around school hours.

c PSS: Perceived Stress Scale.

d FFMQ: Five Facet Mindfulness Questionnaire.

### App Quality and Usability

Overall, the mean caregiver rating of the app was 78.6 (range 55‐100) on the MSUS (>65 is above average usability and >80 is the top 10% of all products tested using the MSUS) [39]. Usability ratings improved from the version used by the first cohort (mean 67.5, SD 22.6) to the second version used by the remaining cohorts (mean 86.2, SD 13.5). Further, 3 of the 11 caregivers rated it below the 65 threshold; all these participants were in the first cohort using the first version of the app. As is common in app development, several issues led to the app being unavailable at times to all cohorts during their program. Specifically, software updates to the mobile app were performed during the intervention period and required resetting of goal reminders or resulted in limited availability of meditations.

### App Usage and Frequency of Meditations

We aimed to support caregivers’ adoption of the app-delivered program by providing reminder notifications and coaching support, to encourage meditations at least twice weekly over 4 months’ time. Further, 1 participant met these criteria, and another nearly met it, but the remainder meditated twice weekly only 5 or less than 5 weeks’ time over the course of the program. On average, they listened for 10.2 (SD 3.8) minutes per week to 20 meditations. Shorter tracks (~5 min in length) were most frequently listened to. The user data suggest that participants, as a group, attempted to listen more often (439 attempts) than they completed podcasts (314 complete or within 30 s of completion), pointing to potential app instability and preference for shorter meditations.

### Levels of Stress and Mindfulness

#### Overview

As a group, participants scored 23 (SD 4.45) on the preintervention measure of stress and 21 (SD 8.23) post intervention. As a reference, the normative mean for this age group, based on a Harris Poll of 2387 respondents, was 12.6 (SD 6.1) on the PSS 10 item [30]. Our reported values are similar to baseline PSS 10 item scores for Bazzano and colleagues’ [18] sample of caregivers (n=66, PSS mean 22.6, SD 6.2), and Conner and colleagues’ [2] (n=67, PSS mean 20.73, SD 7.03). These PSS scores suggest that overall this was a highly stressed group, and these findings are similar to the level of stress found in other studies of caregivers of children with autism or disabilities. At baseline, 3 of 11 participants were over 2 SDs above the normative population mean; the remaining 7 participants were over 1 SD above the mean for population stress levels on PSS [30]. After completion of the 5Minutes4Myself wellness program, more than half of the participants (6 of 11) were within 1 SD of mean for population stress levels [30].

In terms of mindfulness, the preintervention group mindfulness mean was 119.91 (SD 15.49) and ranged from 90 to 146. Post intervention, the mean FFMQ scores were 127 (SD 17.71) with a similar range of 93 to 153. No population norms are available for the FFMQ for comparison to allow us to reflect on the meaningfulness of the participants’ scores.

**Figure 2.**
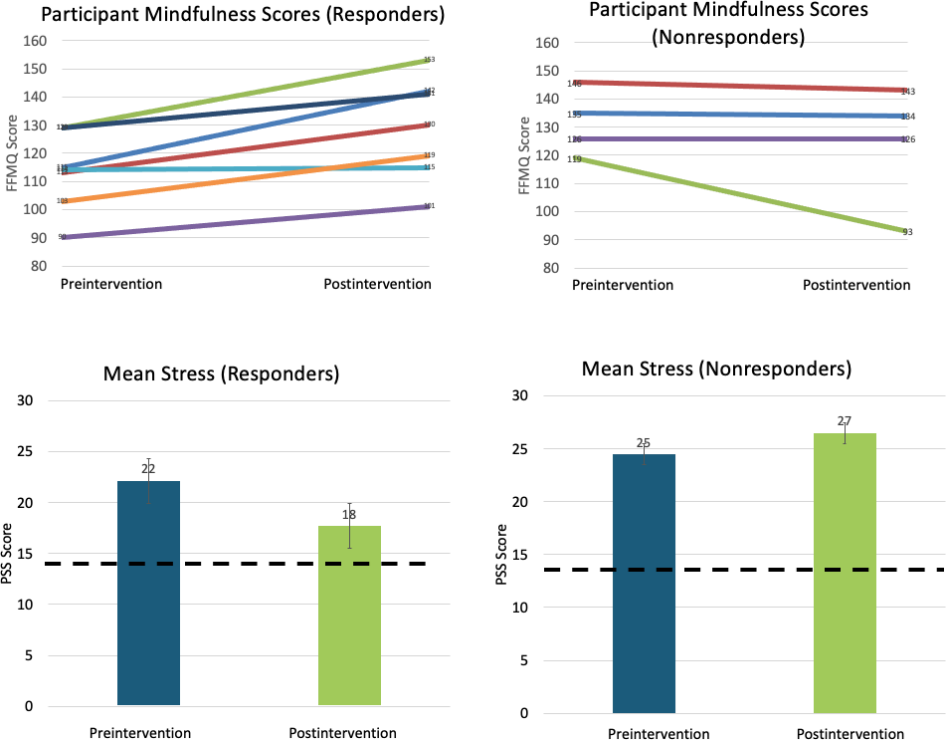
This figure groups scores into responders and nonresponders based on their mindfulness score (FFMQ). Responders’ mindfulness scores on the FFMQ increased from pre intervention to post intervention, as hypothesized for 7 participants. Nonresponders’ mindfulness scores decreased from pre intervention to post intervention for 4 participants. Each line represents 1 participant’s mindfulness score (FFMQ) from pre intervention to post intervention. Corresponding group mean stress (PSS) scores are displayed below. The dotted line indicates a population-based mean for the PSS for this age group [29]. FFMQ: Five Facet of Mindfulness Questionnaire; PSS: Perceived Stress Scale.

To assist in visualizing the data, participants were grouped into mindfulness responders and nonresponders. Responders were identified as individuals whose mindfulness scores improved over the intervention period whereas nonresponders’ mindfulness scores either stayed the same or decreased. Figure 2 displays the individual mindfulness scores (FFMQ) at pre and post intervention and mean stress scores (PSS) for these 2 groups. The small error bars suggest the 2 groups of caregivers are similar to each other in terms of stress.

In terms of individual change in the PSS, individual change calculations ranged from −1.57 to 2.02 (reverse coded so that positive numbers indicated reduced stress). When grouped according to Norman and colleagues’ [38] guidelines, 4 participants experienced worse stress and 7 had reduced stress post participation. This affirms the responder visualization of the data.

#### Changes in Stress and Mindfulness Pre and Post Intervention

A related samples *t* test evaluated whether participants’ PSS mean stress scores were significantly different before and after participating in the wellness program. Preliminary analyses of normality, random selection, and homogeneity of variances were conducted and ensured no assumptions were violated. The group PSS postintervention mean was not statistically and significantly different than the preintervention group mean (*t*_10_=1.20, *P*=.26). The effect size *d* of 0.36 indicates a small to medium effect size. Given the small sample used for this feasibility study, these findings do not clearly indicate whether this change in PSS score was a meaningful difference for all or some of our participants [40].

For mindfulness, a related samples *t* test evaluated whether participants’ FFMQ scores differed following participation in the program. The group mean FFMQ was not significantly different following participation in the program (*t*_10_=−1.57, *P*=.15). However, in this case, the effect size was larger (*d*=0.47), suggesting that a treatment effect might be evident with a larger sample size.

#### Postintervention Stress as Predicted by Change in Mindfulness

Given that 1 effect size suggested some potential treatment effect and the trends noted in responders, a hierarchical multiple regression was used to assess the central question of how change in mindfulness (FFMQ) predicted levels of stress post intervention (PSS), after controlling for preintervention stress levels. Preliminary analyses were conducted and ensured all assumptions of multiple regression were met including linear relationship between variables, normal distribution of residuals, an independent sample, no multicollinearity, and homoscedasticity, despite the small sample size (n=11). The results of this regression are provided in Table 3. These results suggest that 20% of the variance in stress, beyond the 50% of variance accounted for by prestress levels, can be attributed to pre-post changes in the FFMQ (or increases in mindfulness; *P*=.04). Therefore, change in the FFMQ was a significant predictor of postintervention stress levels for these caregivers of children with ASDs.

**Table 3.** Hierarchical regression analysis of predictors of stress.

Predictor variables	Model 1		Model 2	
Preintervention stress (Perceived Stress Scale), B^a^ (SE)	1.29^b^ (0.395)		0.86^b^ (0.36)	
Change in mindfulness (Five Facet Mindfulness Questionnaire), B (SE)	—^c^		−0.28^b^ (0.11)	
*R*^2^/adjusted *R*^2^	0.544/0.493		0.743/0.679	
*F* change in *R*^2^	10.73^b^		6.20^b^	

a B: unstandardized β.

b *P*<.05

c Not applicable.

## Discussion

### Principal Results

This study examined the usability and usage of an app designed to deliver mindfulness meditations and the effects of the mindfulness training on stress experienced by caregivers of children with ASD. The mobile app, as designed and then revised, was found to be above average in usability, especially the second version. Although caregivers did not use the mindfulness meditations as frequently as intended, they did use them on average for 10 minutes per week, a microdose compared to other mindfulness programs for caregivers.

While group differences in pre- and poststress and mindfulness scores were not significant in this analysis, 1 effect size offered tentative support for the impact of the 5Minutes4Myself program on mindfulness. As Cohen [41] suggests, statistical significance and calculations of effect size are useful only in so far as they can be used to interpret the meaningfulness of the score change to the participant [39]. This seems to be supported by the individual level analysis, where 7 of 11 caregivers showed improvement or decreased stress post participation. In addition, this study linked increased mindfulness to a reduction in stress; increases in mindfulness accounted for 20% of the individual variance in postintervention stress scores.

### Limitations

The pre- or postintervention group changes in stress and mindfulness were statistically insignificant. While the individual change analysis did show benefits for some caregivers, this analysis does not identify if any specific characteristics indicate who might be more likely to benefit from the program. Further, the analysis relied on a small sample (n=11). Future analyses of larger samples may help us to better understand the effects of this wellness program on stress and the contribution of the mobile-mindfulness component.

While these preliminary findings support the use of a mobile app in the 5Minutes4Myself wellness program, the developing technology was a significant barrier for some caregivers. Complications with application were a limitation of this study and accounted for lost postsurvey data for 2 participants.

We also did not conduct follow-up measures for this feasibility study. Long-term follow-up measures are needed to examine benefits of participation in the 5Minutes4Myself wellness program within the first- and second-year post participation. Further exploration of the 5Minutes4Myself wellness program will offer greater insight into how to best serve caregivers of children with ASD to support stress reduction and positive well-being outcomes.

### Comparison With Prior Work

When compared to other studies, our caregivers showed similar percentages in reduction of stress (9% mean decrease) and increase in mindfulness (10% increase) as studies assessing larger populations of caregivers who reported statistically significant findings. For example, 2 traditional and more time-intensive MBSR programs found that caregivers’ increased mindfulness was associated with a 7.4% reduction in self-rated perceived stress [19] and that parents and teachers of children with special needs had a 10% increase in mindfulness (as calculated from reported data) and an 8% reduction in stress [18].

Given that increases in mindfulness accounted for 20% of the individual variance in postintervention stress scores, this highlights the importance of mindfulness, be it a small dose, in diminishing stress. Benn and colleagues’ [19] work corroborated that increased mindfulness was key to stress reduction. Using the same stress and mindfulness measures as in our study to assess outcomes of an 8-week MBSR-style training program for parents of children with special needs, Benn and colleagues [19] conducted a mediations analysis. Their analysis identified increased mindfulness as the mediator for positive changes in health and well-being outcomes.

Consistent with previous research that used much larger “doses” of mindfulness, such as those provided in MBSR-based programs [1,12,19], the results of this analysis suggest that a micromindfulness approach is a viable intervention for stress reduction for caregivers of children with ASD. Carmody and Baer’s [42] analysis seems to confirm that shorter mindfulness practices are indeed viable alternatives to the time-intense MBSR programs. They found that in-class hours and treatment effect sizes were not significantly related for their population, suggesting low-dose practices, such as what we used in this program, can be an effective alternative for time-challenged individuals. Lunsky and colleagues [43] also noted that while most of the parents of children with ASD in their study attended only 4 of 6 sessions and reported brief and irregular home practice, these parents still derived psychological benefit with less distress post intervention compared to a control group. Berghoff and colleagues [44] examined dose for college students assigned to either 10- or 20-minute practices of mindfulness for 14 days; they found that significant reductions in stress were independent of the actual time practiced. The total dose for most students was only an hour over 2 weeks. While our caregivers did not listen as often as proposed, using a mobile app for this population appeared to be a feasible delivery system for brief mindfulness training and shorter meditations were more practical for this group. Our approach offered caregivers the flexibility to choose when and where they listened to brief (4‐21 min) mindfulness podcasts while having regular guided practices such as class sessions.

Use of a mobile-delivery system for mindfulness practice opens the opportunity for an intervention that is client-centered and can be more readily incorporated into a participant’s daily routine. Hollis and colleagues [45] suggest that mobile apps have the potential to transform service provision to clients by providing content, monitoring, and access to providers. Yet, findings from a large-scale study using a newly developed mindfulness application (VGZ Mindfulness Coach; VGZ Health Insurer N.V) appear to suggest flexible provision of easily accessible mindfulness content is not sufficient to encourage participation; only 58% of their participants used the application [46]. Engaging clients and clinicians to collaboratively design to meet unmet needs and to assure the usability of the technology is essential to not only develop strongly evidence-based interventions but also assure their adoption by the targeted population [45,47,48].

Most mental health programs for caregivers of children with autism have been delivered solely in person, which may partially account for the high attrition rates ranging from 17%‐45% [49]. We choose to use an app to deliver content to increase the accessibility of this program for caregivers and decrease attrition, which in our small sample was 13%. Preliminary data suggest this mindfulness training provided stress reduction for some of the participating caregivers. In line with Latulippe and colleagues’ [23] findings, we found that in designing an eHealth option for caregivers we needed to attend to user characteristics including (1) limits in financial and personal resources, (2) available access to internet, and (3) users’ comfort in using and managing technology. In addition, for our app it was important to attend to caregivers’ reported reduced problem-solving capacities which were likely due to the ongoing stress and the vigilance of caregiving. Using a collaborative participant-centered approach to app design helped ensure we created a highly usable application that was confirmed via caregiver ratings of above-average usability on the MSUS [10,27,39].

### Conclusions

To our knowledge, this program is the first of its kind to use a mobile app to deliver a wellness program, that included mindfulness meditations, to caregivers of children with autism. The unique contribution of this wellness program, beyond these initial promising findings of increasing mindfulness and reducing stress, is its intentional design to easily fit into caregivers’ challenging and time-demanding daily lives. The 5Minutes4Myself app can be used at anytime and anywhere without the need for traditional in-person mindfulness training. As this study suggests, use of the mobile-mindfulness component may be a feasible intervention for caregivers that allows them to participate in an evidence-based intervention for stress reduction at a time and place that is convenient and possible within their everyday lives.

## Abbreviations

ASD: autism spectrum disorder
FFMQ: Five Facet Mindfulness Questionnaire
MBI: mindfulness-based intervention
MBSR: mindfulness-based stress reduction
MI: motivational interviewing
MSUS: Modified System Usability Scale
PSS: Perceived Stress Scale
